# Retraction: Role of Microvascular endothelial cells on proliferation, migration and adhesion of hematopoietic stem cells

**DOI:** 10.1042/BSR-20192104_RET

**Published:** 2020-07-09

**Keywords:** Cell adhesion, Cell migration, Cell proliferation, Chemotaxis, Hematopoietic stem cells, Microvascular endothelial cells

This Retraction follows an Expression of Concern relating to this article previously published by Portland Press.

This article is being retracted from Bioscience Reports at the request of the authors following receipt of a notification from a reader, alerting the authors to the duplications in Figure 4A as well as duplicated figures from another published article by the same authors in Zhongguo Shi Yan Xue Ye Xue Za Zhi (10.19746/j.cnki.issn1009-2137.2019.02.046).

The authors wish to retract the article. The Editor-in-Chief and Editorial Board agree with the retraction.

